# Winners And Losers In Coronavirus Times: Financialisation, Financial Chains and Emerging Economic Geographies of The Covid‐19 Pandemic

**DOI:** 10.1111/tesg.12433

**Published:** 2020-06-26

**Authors:** Martin Sokol, Leonardo Pataccini

**Affiliations:** ^1^ Department of Geography Museum Building Trinity College Dublin College Green Dublin 2 D02 PN40 Ireland

**Keywords:** Financialisation, financial chains, debt, economic geography, pandemic, crisis

## Abstract

This paper has two interrelated aims. First, it attempts to sketch a preliminary map of economic winners and losers to highlight the emerging economic geographies of the coronavirus pandemic. Second, it aims to explore the links between these emerging economic geographies and the processes of ‘financialisation’, drawing on the concept of ‘financial chains’. Regarding the first aim, the paper argues that the pandemic‐induced crisis will exacerbate social inequalities and deepen uneven development at multiple geographical scales. Regarding the second aim, the paper argues that the ‘financialisation’ perspective in general, and the concept of ‘financial chains’ in particular, provide useful insights into the crisis and its uneven effects, by shedding light on the complex web of flows of value and power relations established/emerging between the prospective winners and losers. It also highlights the prominent role of debt and debt‐based financial chains in shaping economic geographies in times of major global crisis.

## Graphical abstract

This paper highlights the emerging economic geographies of the coronavirus pandemic. It argues that the pandemicinduced crisis will exacerbate social inequalities and deepen uneven development at multiple geographical scales. The paper also aims to explore the links between these emerging economic geographies and the processes of ‘financialisation’, drawing on the concept of ‘financial chains’. It argues that the ‘financialisation’ perspective in general, and the concept of ‘financial chains’ in particular, provide useful insights into the crisis and its uneven effects, by shedding light on the complex web of flows of value and power relations emerging between the prospective winners and losers.

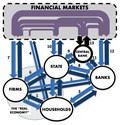

## Introduction

It is becoming clear that the coronavirus pandemic will have unprecedented economic impacts. The evidence is mounting that the world economy is likely to be hit by a shock similar to that of the Great Depression of the 1930s. Who will be the winners and the losers of the coming economic calamity? This paper has two aims. First, it attempts to sketch out a preliminary map of winning and losing actors. In doing so, it aims to highlight emerging economic geographies of the pandemic. Second, the paper aims to explore the links between these emerging economic geographies and the processes of ‘financialisation’ (Aalbers 2016). More specifically, the paper aims to explore the way in which the concept of ‘financial chains’ (Sokol 2017a) can be mobilised to understand the current crisis and its winners and losers.

With regard to the first aim (examined in the following section), we argue that the pandemic‐induced crisis will exacerbate social inequalities and deepen uneven development at multiple geographical scales. The emerging map of winners and losers will most likely further aggravate the existing inequalities in the global economy. In particular, we foresee that the Global North/Global South divide is likely to grow, as will the economic differences *within* these global regions. Indeed, within the Global North, the case of Europe highlights complex economic geographies linked to both north‐south and east‐west divergence, with further fragmentation likely occurring at sub‐national levels.

With regard to our second aim (explored in the third section) we argue that the ‘financialisation’ perspective in general, and the concept of ‘financial chains’ in particular, provide useful insights into the crisis and its uneven effects, both for individual economies and at the international political economy level. We argue that the ‘financial chain’ approach can shed light on the complex web of flows of value and power relations established/emerging between the prospective winners and losers, while also highlighting the prominent role of debt (and debt‐based financial chains) in shaping economic geographies in times of major global crisis.

## Emerging Economic Geographies of the Pandemic

In addition to its significant public health effects, the COVID‐19 pandemic (caused by the novel coronavirus SARS‐CoV‐2) is expected to have unprecedented economic impacts. According to the latest forecast by the IMF, the global economy will face the worst crisis since the Great Depression, surpassing that experienced during the Global Financial Crisis (GFC) (IMF 2020). We argue that the crisis will (re)produce uneven economic geographies within the global economy at multiple scales while also exacerbating social inequalities. We expect that the map of winners and losers will be shaped by the existing socio‐spatial inequalities; by the size and nature of the pandemic‐induced economic shock inflicted on individual economies; by the ability of individual countries to respond to that shock; and by the ability of different economic sectors, regions, countries and whole macro‐regional blocks to recover. We suggest that what is potentially emerging from this, to put it metaphorically, is an economic map with few island‐winners in a vast sea of losers. We first briefly highlight potential winners before exploring the complex map of emerging losers. In particular, we argue that while both the Global North and the Global South will be hit hard, the Global South is likely to suffer more, causing the North/South divide to grow. Furthermore, economic differences are expected to be exacerbated *within* these global regions. In the Global North, we highlight the case of Europe and its complex economic geographies with regard to its north‐south and east‐west divides, both of which may be deepened by the crisis. Further fragmentation is likely to occur at sub‐national levels.

### The winners

The number of economic winners generated by the coronavirus pandemic is likely to be extremely limited and concentrated. Amid unprecedented stock market collapses – with Wall Street experiencing its fastest bear‐market plunge in history (Aslam 2020) – some companies have managed to directly benefit from the health emergency. One example of this is Moderna, a biotech firm based in Cambridge, Massachusetts, whose stock prices soared by 135 per cent upon news that it was making progress in developing a COVID‐19 vaccine (Lee 2020, 2020). A number of other biotechnology, pharmaceutical and medical companies have generated substantial profits since the beginning of the crisis. To this list one can add the companies that have benefited from increased homeworking and imposed lockdowns, such as Zoom Video Communications (USA), Netflix (USA) or Ocado Group (UK), who also saw their stock prices rise rapidly (Table 1).

**Table 1:** Stock price change of selected ‘winner’ firms, January‐April 2020.

Company	Sector	Country	Stock price as of 2 January 2020	Stock price as of 30 April 2020	per cent of change 02 January 2020–30 April 2020
Novacyt	Biotechnology	UK/France	0.18 EUR	4.67 EUR	2494.44
Co‐Diagnostics	Molecular diagnostics	USA	0.91 US$	11.34 US$	1146.15
Novavax	Biotechnology	USA	4.49 US$	18.13 US$	303.79
Inovio Pharmaceuticals	Biotechnology	USA	3.21 US$	12.03 US$	274.77
Kawamoto Corporation	Manufacturing of medical supplies	Japan	455 JPY	1335 JPY	193.41
Moderna	Biotechnology	USA	19.23 US$	45.99 US$	139.16
Teladoc Health	Telemedicine and virtual healthcare	USA	83.26 US$	164.59 US$	97.68
Zoom Video Communications	Communications technology	USA	68.72 US$	135.17 US$	96.70
Tianjin Teda	Conglomerate	China	3.75 CNY	7.31 CNY	94.93
Regeneron Pharmaceuticals	Pharmaceutical	USA	373.35 US$	525.88 US$	40.85
Pharma Mar	Biopharmaceutical	Spain	3.89 EUR	5.45 EUR	40.10
Amazon.com	Conglomerate	USA	1898.01 US$	2474 US$	30.35
Gilead Sciences	Biotechnology	USA	65.23 US$	84 US$	28.78
Ocado Group	Internet Retail	UK	1259.50 GBP	1604.50 GBP	27.39
Netflix	Media	USA	329.81 US$	419.85 US$	27.30
Hansoh Pharmaceutical Group	Pharmaceutical	China	25.25 HKD	30.10 HKD	19.21

The benefits of the pandemic have been so concentrated that one can even identify individuals who are among the winners. According to Forbes reports, Stéphane Bancel, the CEO of Moderna, has become a billionaire overnight after the company announced that it was expected to begin human trials for its COVID‐19 vaccine (Tognini 2020). Likewise, several other businessmen and businesswomen have benefited from the pandemic, such as Wee Chai Lim, Chairman and Co‐Founder at Top Glove Corporation BHD (Malaysia) (Lee & Raghu 2020); Zhong Huijuan, the chairwoman of Hansoh Pharmaceutical and her husband, Sun Piaoyang, the chairman of Jiangsu Hengrui Medicine (China), just to name a few (Cuccinello 2020). More broadly, shareholders/investors of the benefiting companies are among the few economic agents who have made large gains amid the global economic collapse.

Beyond this, other big winners of the pandemic will likely be private equity funds, hedge funds and/or institutional investors who will be able to take advantage of the stock market collapse to buy valuable assets at extremely low prices, as was the case in the last crisis (see Beswick *et al*. 2016). We expect that the current crisis will produce a range of similar opportunities across sectors and asset classes (see Fujita 2020; Reuters 2020b; Sharma & Tripathy 2020; Smith & Platt 2020; for emerging examples).

### The global economy in crisis

It is becoming increasingly clear that apart from a few isolated sectors and actors (such as those mentioned above) the entire global economy will experience a major downturn. The IMF (2020) predicts that the world economy as a whole will contract sharply – by 3 per cent in 2020 – making the ‘Great Lockdown’ a bigger economic calamity than the one caused by the 2008 GFC. However, we argue that this contraction will not be felt evenly across individual economies (although not necessarily exactly the way the IMF predicts). Indeed, there are significant differences in the effects of the crisis, in exposure levels and in response capacity between countries and whole macro‐regions. The combination of these factors will shape the economic geographies of the pandemic – on top of substantial pre‐existing inequalities.

One of the factors that is hard to predict at the moment is how long individual economies will remain under lockdown. The OECD (2020a) forecasts that for each month of containment, there will be a loss of 2 per cent in annual GDP growth, with some sectors, such as tourism and hospitality, experiencing a much larger decline, reaching up to 70 per cent. Big losses are also expected in related sectors, for instance in travel (airlines) and oil‐related industries. This will hit the countries, regions, cities and workers most dependent on these sectors the hardest. It remains to be seen exactly how bad the economic damage is going to be. The IMF (2020, p. 1) itself admits that there is ‘extreme uncertainty around the global growth forecast’. Regardless, the IMF (2020) expects that in 2020 advanced economies will be hit hard and will fall by 6.1 per cent, before recovering in 2021. These and other predictions may yet prove to be too optimistic.

One of the most striking, and immediately visible, aspects of this economic calamity is a dramatic increase in unemployment. The speed and the size of the labour market collapse seem unprecedented. Within the first month of the COVID‐19 emergency in the US, 22 million workers lost their jobs (DoL 2020), with the unemployment rate nearing 18 per cent (Lambert 2020), breaking post‐War and post‐Depression records. In the UK, the Institute for Employment Studies estimated that up to 2 million people lost their jobs within the first month of the crisis, with unemployment jumping from 3.9 per cent to 7.5 per cent, already surpassing the peak of the last recession (Wilson *et al*. 2020). It is likely that these figures will grow further, disproportionately hitting low‐pay and precarious workers, and driving social inequality to new highs.

**Figure 1:**
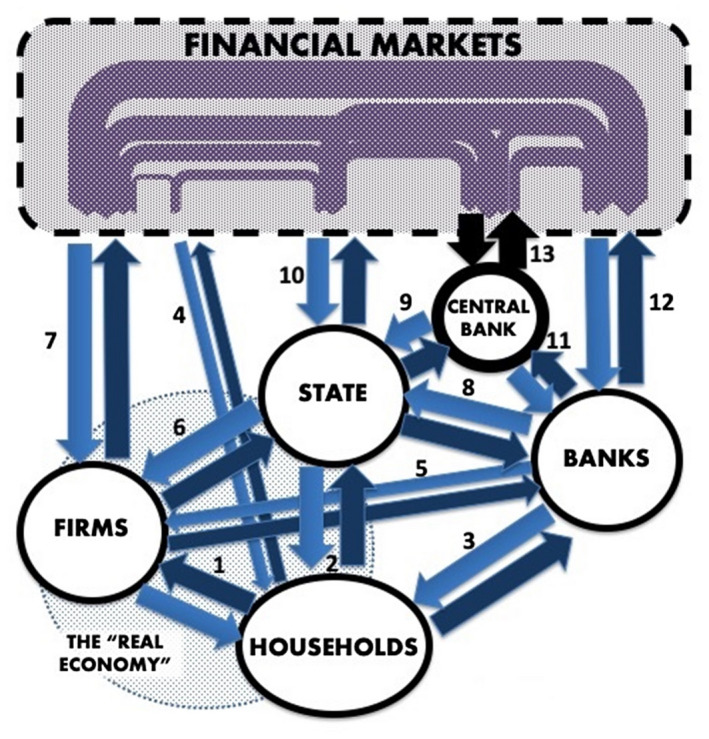
Financial chains. [Colour figure can be viewed at wileyonlinelibrary.com] *Source:* Adapted from Sokol (2020). *Note:* chain 1: labour value/ wages; chain 2: personal taxes/ welfare payments; chain 3: bank lending to households/ debt repayments by households; chain 4: investments by households/ returns; chain 5: bank lending to firms/ debt repayments by firms; chain 6: corporate taxes/ state subsidies and bailouts; chain 7: investments/ returns; chain 8: government borrowing from banks/ repayments; chain 9: government borrowing from the central bank/ repayments (or monetary financing); chain 10: government borrowing from financial markets/ repayments; chain 11: bank borrowing from the central bank/ repayments; chain 12: bank investments and borrowing via financial markets; chain 13: central bank interventions on financial markets (e.g. quantitative easing).

The realisation that this crisis is threatening the entire economic edifice has led to some extraordinary policy responses (see Sokol 2020). Both the US and the UK, perhaps the two most neo‐liberalised/financialised^1^ among the advanced countries, have responded with massive fiscal interventions. The US Government’s US$2.1 trillion rescue package seems to be the biggest economic stimulus in history (see also Buiter 2020). The UK, meanwhile, is spending some 7.5 per cent of GDP on coping mechanisms (Inman 2020). In addition to such unprecedented fiscal interventions, we have also witnessed a dramatic mobilisation of monetary policy instruments. Indeed, this massive government spending has been matched by colossal interventions from central banks. Apart from slashing interest rates, both the Fed and the Bank of England have been injecting liquidity into financial markets though quantitative easing (QE) and extending credit lines to their governments in order to cover their extraordinary expenditure (Giles & Georgiadis 2020; Marte 2020). It is likely that, without such actions, financial markets would have collapsed completely – demonstrating just how central the central banks really are in sustaining contemporary financialised economies and their ‘financial chains’ (see Figure 1 and the third section).

There are, of course, major issues related to money‐creation programmes such as QE. The monetary interventions by the Fed and other central banks certainly helped to contain the previous crisis of 2008. However, it did so by helping to build an unprecedented pile of debt that is now weighting on the whole global economy, making it a lot more vulnerable to the pandemic‐induced economic shock (see also below). According to the OECD (2020b), at the end of December 2019 the global outstanding stock of non‐financial corporate bonds in advanced economies amounted to US$13.5 trillion, twice the level of December 2008 in real terms. In the US alone, non‐financial corporate debt totalled US$6.6 trillion at the end of 2019, twice the amount before the GFC (Cox 2020). Much of this debt has been created on the back of QE and ultra‐loose monetary policy applied by the Fed in the aftermath of the 2008 crisis (see Plender 2020). The problem is that a large portion of this debt keeps alive the so‐called ‘zombie’ banks and companies that are extremely vulnerable to the slightest economic shock (Plender 2020). ‘Zombie firms’^2^ now appear to represent about 16 per cent of all the publicly traded companies in the US, and more than 10 per cent in Europe (Banerjee & Hofmann 2018), which has major implications for financial stability. The issue of debt is further amplified at the global level. At the end of 2019, total world debt reached US$253 trillion, equivalent to 322 per cent of global GDP, the highest ever recorded level (IIF 2020a). The levels of debt are particularly worrying for the Global South, to which we now turn.

### The Global South

The coronavirus pandemic poses a number of serious threats to developing and emerging countries (DECs) in the Global South. The quarantine measures applied in many of these countries to contain COVID‐19 contagion have led to serious economic collapses, sharp rises in unemployment and a significant reduction in state revenues (UNCTAD 2020). This creates an additional challenge because, unlike the developed economies of the Global North, many DECs have much weaker fiscal capacity to implement large stimulus packages (or to apply effective monetary policies). The governments in the Global South also have less resources to support laid‐off workers. The situation will be even worse for those depending on self‐employment or informal work – which represent a large part of labour in Latin America, Asia and Africa, and cannot be carried out remotely (ILO 2020). The collapse of the informal economy may not be accurately captured in official statistics (or IMF predictions). The crisis is likely to hit the weakest sections of society the hardest. By the time the pandemic is over, half the world's population may be living in poverty (Oxfam 2020), the vast majority of them in the Global South. Prospects for the Global South are not very encouraging.

The global economic slowdown expected for 2020 and beyond will significantly reduce the demand for exports from DECs. Moreover, the decline in export volume will be accompanied by sharp falls in energy and commodity prices, which still make up most of the goods that many countries from the Global South export. According to UNCTAD (2020), developing countries as a whole (excluding China) will lose nearly US$800 billion in terms of export revenue in 2020. Moreover, other contributing items in their current accounts, such as remittances, are likely to decline as well. Thus, the fall in hard currency inflows will put further pressure on the exchange rates, while many countries in Latin America and other developing regions have already experienced devaluations (see below). This means not only that their exports will fall in volume and value, but also that the terms of trade *vis‐à‐vis* the advanced economies will deteriorate dramatically, producing a relative impoverishment of these economies in the international markets.

We argue that, on top of this, a major source of weakness for developing economies in the pandemic crisis will be their financial position. Over the past decade, the global liquidity promoted by central banks in the Global North (such as the Fed’s policies mentioned above) has contributed to a systematic build‐up of financial and debt vulnerabilities in countries of the Global South. The pandemic has triggered a flight to safety that caused record capital outflows from DECs. According to the International Institute of Finance, in March 2020 alone, Emerging Markets securities suffered around US$83.3 billion in outflows (IIF 2020b), which is more than three times the portfolio outflows experienced by the same countries during the GFC of 2008. In turn, these capital flights have already triggered widening spreads and large currency depreciations.

A critical vulnerability for developing and emerging economies relates to debt. Total debt in DECs reached a record US$55 trillion after the ‘largest, fastest, and most broad‐based’ debt wave in half a century (World Bank 2019; Ayhan Kose *et al*. 2020), with the debt‐to‐GDP ratio surging by 54 percentage points between 2010 and 2018 to a record high of 168 per cent. The total external debt of DECs reached 26 per cent of GDP in 2018, but if China is excluded, the external debt stood at 35 per cent of GDP. It is important to note that government debt accounted for almost three‐fifths and private debt for little over two‐fifths of the total debt increase between 2010 and 2018 (UNTACD 2020). This means that both the public and private sectors in DECs will face increasing pressure on debt service in the coming years, especially those denominated in foreign currencies, in a context in which rolling over that debt seems unlikely. This situation, coupled with the declining export earnings, widening spreads and sharp currency devaluations mentioned above, will seriously jeopardise DECs’ ability to repay their debts and pay for the imports necessary to keep their economies running. Furthermore, as suggested by Joseph Stiglitz (2020), many DEC governments will face a dilemma between paying off their external debts or using these funds to fight the domestic health and economic effects of Covid‐19. There is a serious danger that the situation could descend into a major global debt crisis (see also Ghosh 2020; Roos 2020).

It remains to be seen what winners and losers such a scenario will produce but, unless some global rescue efforts are put in place, the crisis is likely to hit the weakest economies the hardest. It also remains to be seen how China and India, the two biggest emerging economies and both intimately connected to the fortunes of the global economy, will withstand the global downturn.^3^ What can be said already, however, is that the emerging map of winners and losers will most likely aggravate inequalities in the global economy. Taking into consideration the factors discussed above, it looks likely that the broad gap between the Global North and Global South will widen. However, we would like to highlight the fact that economic differences *within* these global regions are also likely to grow. This can be illustrated by the case of Europe, which we examine in turn.

### Economic geographies of pandemic Europe

Europe provides a fascinating example within the Global North of how economic geographies of the pandemic will be shaped by pre‐existing inequalities; by the intensity of the pandemic‐induced economic shock inflicted on individual economies; by the ability of individual countries to respond to that shock; and by their ability to stage an economic recovery. Of crucial concern here is the core‐periphery dynamic, or more specifically, the divergence between northern Europe and southern Europe. Describing Europe in terms of north‐south divide is, of course, an oversimplification of a much more complex economic geography of Europe (see Sokol 2001). But the fact is that significant economic differences between the ‘core’ (Germany, Belgium, the Netherlands, etc.) and the southern ‘periphery’ (Portugal, Spain, Italy, Greece) remain despite decades of European integration. The Global Financial Crisis of 2008 and the subsequent sovereign debt crisis in Europe have brutally exposed, and further deepened, these differences. EU‐imposed austerity may have stabilised the eurozone and allowed the core to recover, but it left the southern periphery weaker and more vulnerable.

Indeed, in 2019 Germany’s output was approximately 15 per cent higher than in 2007; Belgium’s was 13.5 per cent higher and the Netherlands’ was up by 12 per cent (World Bank 2020). By contrast, the Italian GDP in 2019 was nearly 6 per cent lower than in 2007 and the Greek GDP was, astonishingly, nearly 25 per cent lower, while the 2019 outputs of Spain and Portugal were only about 4.5 per cent and 2.5 per cent above their respective pre‐crisis levels (World Bank 2020). The contrasts in sovereign debt levels between the north and the south are equally stark. Germany's sovereign debt‐to‐GDP ratio stands at approximately 62 per cent and that of the Netherlands at 52 per cent. Meanwhile, Italy’s sovereign debt burden is equivalent to almost 135 per cent of its GDP and Greece's exceeds 185 per cent of GDP. The Italian and the Greek banking sectors are also among the most fragile in Europe, with high ratios of non‐performing loans (Reuters 2020a). Stark differences in economic performance are also reflected in unemployment levels. Greece, Spain and Italy have the highest unemployment rates in the EU, reaching 16 per cent, 14 per cent and 10 per cent, respectively, in contrast to Germany’s 3.2 per cent and the Netherland’s 2.9 per cent (Eurostat 2020).

All these differences are about to be dramatically exacerbated by the crisis. Extending the analogy made by Roos (2020) in connection to Italy, it is a ‘cruel irony’ that the southern periphery, being the weakest economically, was also hardest hit by the coronavirus. Furthermore, on top of everything else, the economic and employment structure of the southern periphery makes it extremely vulnerable to the current crisis. Tourism accounts for a significant proportion of employment in Greece (over 25%), Portugal (over 20%) as well as Italy and Spain (nearly 15%), while these countries are also more dependent on small businesses which may have a harder time surviving the crisis or taking advantage of any employment protection schemes (Arnold & Dombey 2020). The IMF forecasts that unemployment rates are expected to increase significantly in 2020, peaking at over 20 per cent in Spain and Greece, about 14 per cent in Portugal, and 13 per cent in Italy, compared to 4 per cent in Germany (IMF 2020).

It is expected that the eurozone as a whole will be hit hard by the crisis, with the block’s GDP falling by as much as 7.5 per cent in 2020 (IMF 2020). However, it is widely expected that the fall in the southern periphery will be more pronounced, while its subsequent recovery will be weaker (see also Münchau 2020). Thus, the uneven economic impact of the crisis combined with the uneven recovery, playing out on an already uneven terrain, will drive the north‐south divide to new levels.

In the absence of a strong EU‐wide supporting mechanism (such as ‘eurobonds’^4^), the diverging economic fortunes of the southern periphery can have unpredictable consequences. For example, Italy’s falling GDP combined with rising debt could push its debt‐to‐GDP ratio from the current 135 per cent to between 160 per cent and 180 per cent, raising doubts about Italy’s solvency and potentially leading to a default (Münchau 2020). A sovereign debt default by Italy or any other country in the southern periphery could rip the eurozone, and the EU, apart. The prospect of a disintegration of the EU is of crucial concern to member states in the ‘eastern’ periphery.

### East‐Central European dimension

It is worth remembering that one of the key reasons for post‐socialist countries in East‐Central Europe to join the EU was to catch‐up economically with their Western European counterparts. Closing the economic gap between the ‘West’ and the ‘East’, however, has been proving challenging. Deep ‘transition’ recessions in the early 1990s that followed the collapse of state‐socialism have meant that the economic divide between the Western and Eastern halves of Europe has actually grown larger, as have differences within the East itself. Through the combination of old historical legacies and uneven effects of transformation processes, Central and Eastern Europe has established itself as a European ‘super‐periphery’ while itself internally displaying increasing divergence of economic fortunes (Sokol 2001). From the ‘varieties of capitalism’ perspective, the economies of East‐Central Europe (ECE) have been described as ‘dependent market economies’ (Nölke & Vliegenthart 2009) – their productive capacity became dominated by Western European FDI and their financial systems became heavily dependent on Western European banking groups. The economic fortunes of the entire region have thus became inextricably linked to those of Western Europe. In the early 2000s this dependence helped to foster a remarkable economic revival and rapid catching‐up of many ECE countries. Part of this boom was related to increasing financialisation, not least through the Western banks that were instrumental in rapidly expanding credit across the region, on the back of newly created ‘financial chains’ (Sokol 2017b).

However, for many people and places, all this came to a rather abrupt end when the GFC hit in 2008. The economic damage caused by the crisis was enormous (e.g. see Smith & Swain 2010), in many cases wiping out any gains made in the previous decade. The impacts, however, were unevenly distributed, as was the recovery. Poland, for example, emerged as the only country in the entire European Union to avoid a recession, while Baltic countries experienced deep falls and took nearly a decade to get back to pre‐crisis level (Pataccini & Eamets 2019; Pataccini 2020). For many ECE countries, even the best performing ones, the prospects of fully converging to GDP levels of Western Europe appear as distant as ever.

Following the ‘transition’ recession of the 1990s and the GFC of 2008, the pandemic‐induced crisis of 2020 will be the third major economic calamity in as many decades and perhaps the biggest one yet. Indeed, the impact could be explosive. Economically, the crisis will be playing out on an already uneven economic terrain and is likely to have uneven impacts. The exact contours are hard to predict at this time – but much will depend on the way in which economic and financial links with Western Europe will unfold. The exposure of ECE economies is both to the northern core (for instance via the German automotive industry) and to the southern periphery (Italian banking). It remains to be seen in what ways the existing ‘financial chains’ will survive, be disrupted, transformed and/or replaced. One way or another, the crisis is likely to exacerbate some existing inequalities while also creating new ones – both between and within countries. Indeed, social inequalities and regional disparities within ECE countries are likely to get worse, with the impact being felt all the way down to urban and local levels. Crucially, the weakest ECE states may find it even harder than Italy or Spain to borrow money to fund their recovery (let alone to address growing social and regional disparities), which will only make their situation worse. This leads us to consider the operation of the financial system and its uneven impacts under the conditions of financialisation.

## Crises, Financialisation and Financial Chains

### Financialisation and financial chains

Financialisation, a shorthand for the growing power of finance over societies and economies, has been recognised as the key feature of contemporary capitalism. In a broad sense, financialisation can be defined as ‘the increasing dominance of financial actors, markets, practices, measurements, and narratives at various scales, resulting in a structural transformation of economies, firms (including financial institutions), states, and households’ (Aalbers 2016, p. 2). Building on this, we would like to draw attention to the ways in which firms, financial institutions, states and households in a financialised economy are interconnected through a myriad of ‘financial chains’ (Figure 1). Financial chains could be defined as ‘channels of value transfer (between people and places) and as social relations that shape socio‐economic processes and attendant economic geographies’ (Sokol 2017a, p. 679). Credit‐debt relationships could be considered as prime examples of ‘financial chains’ in a financialised economy. Such credit‐debt ‘financial chains’ are linking (chaining) households, financial institutions, enterprises, nation‐states and financial markets together, with significant implications for the economic fortunes of localities, regions, and whole nations. The pandemic‐induced crisis is a perfect opportunity to explore the operation of financial chains and their role in creating economic winners and losers.

### Crisis and financial chains in a financialised economy

Figure 1 represents a simplified illustration of the web of financial chains in an abstract financialised economy. Key economic players entangled in these chains are characterised as households (workers and consumers), firms (productive enterprises), banks (commercial banks and other credit institutions serving firms and households), the state, the central bank and the ‘financial markets’. At first glance it may appear that Figure 1 depicts financial flows in any capitalist economy. However, in line with the above definition of financialisation, we would like to highlight the growing economic importance of ‘financial markets’ and a plethora of financial actors that directly make up those markets – investment banks, pension funds, mutual funds, insurance companies, private equity funds, hedge funds, etc. Some of these financial market players increasingly act as a ‘shadow banking’ system (Gabor 2016), underlying a shift from traditional bank‐based finance to market‐based finance. A main feature of financialised capitalism is precisely the growing power of financial markets. As critics (e.g. Pettifor 2014) argue, this is at the expense of the productive economy (often also referred to as the ‘real economy’), while also making the system more susceptible to crises.

The current crisis did not start as a financial crisis, but it may end up as one. The pandemic‐induced shutdown of the economy creates a major disruption at the heart of the productive economy: households (workers) under lockdown can neither produce nor consume. The critical value chain of the ‘real economy’ between firms and households (chain 1) is thus being disrupted, creating a tsunami of knock‐on effects for the rest of the economic system: personal taxes (chain 2); debt repayments by households (chain 3); debt repayments by firms (chain 5); corporate taxes (chain 6); dividends to investors via financial markets (chain 7); etc. (see Figure 1). The state can play a stabilising role and support firms, households and banks, but it needs to borrow from somewhere in order to do so. Traditionally, in times of crisis, the state would try to borrow from financial markets (chain 10) via government bonds to finance its growing budget deficit. In the current global downturn, most countries are likely to experience a version of this scenario. But the key point here is that some countries will be much less able to cope with it than others. Indeed, while the countries deemed economically strong will be able to continue to borrow at ultra‐low or even negative interest rates (as is the case for Germany), many others will see their borrowing costs rising to unsustainable levels. In this way the financial markets and attendant debt‐based financial chains will further exacerbate existing economic disparities and will shape the map of winners and losers (see also below).

There is only one player that can potentially alter the above dynamics for any given economy: the central bank (Figure 1). This crisis has demonstrated yet again just how central the central banks really are for the financialised economy and its survival (see also Lapavitsas & Mendieta‐Muñoz 2016). Apart from setting interest rates (thus influencing the cost of debt for all participants), the central bank can directly intervene in the financial markets (chain 13), for example through quantitative easing. In addition to this, the central bank can of course directly lend funds to the banks (chain 11) and, more controversially, to the state (chain 9). With regard to the latter, the unprecedented nature of the current crisis has reignited the debate about ‘monetary financing’ or ‘helicopter money’ (e.g. Sandbu 2020), which would involve simply creating electronic money and passing it directly onto the state or households. It remains to be seen to what extent major central banks will adopt such unconventional policies.^5^ What is increasingly clear, however, is that central bank actions have significant implications for the international political economy, to which we now turn.

### Crises, financial chains and the international political economy

There are several issues related to central bank interventions that deserve attention as they have implications for the emerging economic geographies of the pandemic. First, it is useful to remember that not all central banks are born equal. Indeed, some central banks appear to have unlimited ‘firing power’, while interventions by other central banks look more like ‘fire‐fighting’. The latter group of central banks could be mostly found in the Global South or in the peripheries of the Global North (for instance in East‐Central Europe). Their capacity to support their domestic economies in times of crisis is somewhat limited, affecting any post‐crisis recovery.

Second, even the most robust interventions by the most powerful central banks in the Global North (such as those undertaken by the Fed) do not actually ‘resolve’ the crisis. What they do instead is that they help to create new debt, to ‘resolve’ the old one. Indeed, while the central bank’s purchases of assets in the secondary market inject liquidity that allows to make new loans, low interest rates ensure that borrowing is cheap, thus encouraging more debt.^6^ From the ‘financial chains’ perspective, this basically creates new debt‐based financial chains that stretch value flows (and the power relations that go with them) in *time*. Central banks are thus effectively helping to postpone the crisis into the future (cf. Harvey 2006). In doing so, they are also sowing the seeds of the next crisis (cf. Plender 2020). Third, such interventions by central banks have significant impacts beyond their domestic economies. Indeed, the newly created debt spills over into a wider international political economy (see below). In other words, such debt‐based financial chains (with attendant value flows and power relations) are stretched in *space*, across international borders.

All of these three aspects are now in play during the pandemic‐induced crisis and will shape the emerging map of winners and losers. Indeed, we have seen the powerful interventions (including renewed QE) by central banks around the world, although the Fed’s action was by far the most robust (Boesler & Takeo 2020). The problem with this, as already alluded to above, is that it only creates another wall of debt – on top of that created after the last crisis. Indeed, it has been estimated that some US$11 trillion has been injected into the economy via QE between 2008 and 2018 in the US, Japan and the eurozone alone, and that the total figure may be US$15 trillion, that is, about 20 per cent of the global GDP, making central banks key bond holders (see Fernandez *et al*. 2018). The newest round of QE will further expand this debt mountain. So, yet again, the crisis will not actually be ‘resolved’, only pushed further into the future. Of course, we may choose not to worry about the future (since, as Keynes would remind us, in the long run we are all dead anyway), but it is also possible that, ultimately, some of these debts will have to be reconciled. It is hard to tell who will bear the brunt of the cost (see also Plender 2020) but one can expect that the least powerful actors may end up paying the heaviest price.

We argue, from an economic geography perspective, that many developing and emerging economies may eventually suffer the most. Indeed, there is a good body of evidence (Lim *et al*. 2014; Caldentey 2017; Fernandez *et al*. 2018) about important, and mostly negative, impacts that QE policies in the Global North have on economies in the Global South. There is a growing recognition that a significant portion of funds released by QE flows into the Global South as credit‐debt via international bond markets (see also Fernandez & Aalbers 2019). The figures show that the stock of international bonds from Latin America and the Caribbean region increased from US$297 billion in 2009 to US$871 billion in 2019, whereas in the Asia‐Pacific region it rose from US$253 billion to US$850 billion over the same decade (BIS 2020). This indicates a dramatic expansion of debt‐based financial chains reaching out to the Global South. One major problem with this, as Fernandez *et al*. (2018, p. 7) note, is that such capital flows may go into reverse, ‘leaving behind a stock of unpayable debt’.

The dramatic build‐up of debt in the Global South did not go unnoticed. Back in December 2019, the World Bank (2019) warned of serious risks created by the largest wave of debt accumulation in the countries of the Global South in the last 50 years. In other words, the Global South was already in an extremely vulnerable position, even before the coronavirus pandemic hit (see also Ghosh 2020; Roos 2020). The pandemic has now triggered a flight‐to‐quality stampede from the Global South to more profitable alternatives (such as shares in leading‐edge companies based in advanced economies) or safer options (such as US Treasury bills). Financialisation is a crucial feature of these movements, not only because it promotes the liberalisation and deregulation of capital mobility on a global scale (Epstein 2005), but also because it enables the realisation of profit even without being related to the creation of surplus value (Lapavitsas 2013; Lapavitsas & Mendieta‐Muñoz 2016).

This outflow of capital from the economies of the Global South will further complicate their already problematic financial position. The debt that Global South economies have accumulated since the last crisis has been built on financial chains that systematically transfer value to advanced economies, while it has also substantially increased the Global South’s vulnerability to external shocks. The shock produced by the pandemic could trigger a ‘debt deluge’ (Ghosh 2020; Roos 2020), putting the Global South’s economies on the brink of an unprecedented debt crisis.

All this highlights the point that unfolding processes of financialisation (and their effects) must be understood in a very geographical way. Building on a notion introduced by Fernandez and Aalbers (2019) who see subordinated financialisation in the Global South as a contemporary form of uneven and combined development, we argue that inserting financial chains into the analysis may help to further elucidate the emerging economic geographies in crisis times. Such an approach also highlights the centrality of debt in shaping prospective winners and losers.

## Concluding Remarks

This paper has attempted to address two interrelated aims. First, we have attempted to sketch out the emerging economic geographies of the coronavirus pandemic, highlighting possible winners and losers. Second, we have aimed to explore the links between these emerging economic geographies and the processes of ‘financialisation’ (Aalbers 2016), while drawing on the concept of ‘financial chains’ (Sokol 2017a).

With regard to our first aim, we have argued that the pandemic‐induced crisis will exacerbate social inequalities and deepen uneven development at multiple geographical scales. The emerging map of winners and losers will most likely be conditioned by the existing imbalances in the global economy. In particular, we foresee that the Global North/Global South divide is likely to be exacerbated, with the Global South potentially facing unprecedented debt crisis. The vulnerabilities built since 2008 will be exposed to the limit. Major disruptions to international financial chains can be expected (unless some coordinated global action is taken). Simultaneously, it is most likely that the economic differences *within* both the Global North and the Global South will grow. Taking Europe as an example, we have highlighted the fact that the pandemic‐induced crisis is likely to dramatically deepen the disparities between the northern ‘core’ and the southern ‘periphery’ and between the west and the east, as well as within the east. Further fragmentation is likely to occur at sub‐national levels unless bold policies are implemented to counter this.

With regard to our second aim we have argued that the ‘financialisation’ perspective in general, and the concept of ‘financial chains’ in particular, provide useful insights into the crisis and its effects. Indeed, the ‘financial chains’ approach helps to highlight the interdependencies between various economic actors, including ‘contagion’ mechanisms of the current crisis both within individual economies and at the international political economy level. In doing so, the prism of ‘financial chains’ helps us to shed light on the position of prospective winners and losers in a complex web of flows of value and power relations in financialised/financialising economies. It also draws attention to the centrality of debt and the role of debt‐based financial chains which are likely to be created, disrupted or transformed by the current crisis. In turn, these financial chains will play an active role in shaping economic geographies at a time of major global crisis.

## Notes

1 In line with the definition of financialisation provided in the third section, we see both the US and UK economies as being heavily dependent on financial markets, actors and practices.

2 A ‘zombie’ or ‘living dead’ firm is a company that is unable to cover debt servicing costs from current profits over an extended period of time (Banerjee & Hofmann 2018).

3 At the time of writing, the IMF (2020) predicts that China and India will basically be the only two major economies registering a positive growth in 2020, skewing the average for the ‘Emerging Markets and Developing Economies’ group as a whole. It remains to be seen how accurate such predictions will be.

4 See Sokol (2020) for a brief summary of the struggles involved.

5 It was reported in early April 2020 (Giles & Georgiadis 2020) that the UK had become ‘the first country to embrace the monetary financing of government’ to fight the economic fallout of the pandemic.

6 The European Central Bank is explicit that its QE programme of buying bonds from banks aims at increasing the price of these bonds and creating money in the banking system, causing interest rates to fall and loans to become cheaper, so that ‘businesses and people are able to borrow more’ (see ECB’s explainer of ‘How quantitative easing works’ – https://www.ecb.europa.eu/explainers/show‐me/html/app_infographic.en.html).
